# Characterization of a lung epithelium specific E-cadherin knock-out model: Implications for obstructive lung pathology

**DOI:** 10.1038/s41598-018-31500-8

**Published:** 2018-09-05

**Authors:** S. Post, I. H. Heijink, L. Hesse, H. K. Koo, F. Shaheen, M. Fouadi, V. N. S. Kuchibhotla, B. N. Lambrecht, A. J. M. Van Oosterhout, T. L. Hackett, M. C. Nawijn

**Affiliations:** 1 University of Groningen, University Medical Center Groningen, Department of Pathology & Medical Biology, laboratory of Experimental Pulmonology and Inflammation Research (EXPIRE), Groningen, The Netherlands; 2 University of Groningen, University Medical Center Groningen, GRIAC Research Institute, Groningen, The Netherlands; 3 University of Groningen, University Medical Center Groningen, Department of Pulmonology, Groningen, The Netherlands; 4 University of British Columbia, Centre for Heart and Lung Innovation, Department of Anesthesiology, Pharmacology and Therapeutics, St. Paul’s Hospital, Vancouver, British Columbia Canada; 5 Laboratory of Immunoregulation and Mucosal Immunology, Department for Molecular Biomedical Research, Inflammation Research Centre (IRC), Ghent, Belgium; 6 Department of Pulmonary Medicine, Ghent University, Ghent, Belgium; 7 Department of Pulmonary Medicine, Erasmus University Medical Center Rotterdam, Rotterdam, The Netherlands

## Abstract

The airway epithelium regulates responses to aeroallergens, acting as a physical and immunological barrier. In asthma, epithelial barrier function and the expression of adherens junction protein E-cadherin is compromised, but it is unknown whether this is cause or consequence of the disease. We hypothesized that airway epithelial loss of E-cadherin is a critical step in the development of manifestations of asthma. We generated a transgenic mouse model with conditional loss of E-cadherin in lung epithelial cells at birth and onwards. We observed normal lung development at the time of birth in mice lacking E-cadherin in the lung epithelium. However, E-cadherin deficiency led to progressive epithelial damage in mice growing into adulthood, as evidenced by airway epithelial denudation, decreased zonula occludens (ZO)-1 expression, loss of ciliated cells, and enlarged alveolar spaces. In addition, spontaneous goblet cell metaplasia with mucus production was observed. These epithelial changes were accompanied by elevated levels of the epithelial-derived chemokine CCL17, infiltration of eosinophils and dendritic cells, and mucus production. In conclusion, loss of E-cadherin induces features in the lung reminiscent of those observed in asthma, indicating that the disruption of E-cadherin-mediated cell-cell contacts may play a key role in the development of asthma manifestations.

## Introduction

The airway epithelium forms a structural and immunological barrier against environmental insults, such as inhaled allergens, viruses and particular matter. The pseudostratified airway epithelial layer that lines the conducting airways is composed of basal/progenitor epithelial cells, columnar ciliated cells and mucus secreting cells, of which the latter two are responsible for mucociliary removal of inhaled environmental particulates. Epithelial barrier function is maintained by formation of tight and adherens junctions. Tight junctions are comprised of proteins such as occludin, zonula occludens (ZO)-1 and claudins, and maintain a size- and ion selective barrier, regulating the permeability of the epithelium^1,2^. Adherens junctions, which contain the transmembrane protein E-cadherin, are critical for maintaining apical-basolateral polarization and adhesion to neighboring cells^3^. E-cadherin-mediated contacts are thought to provide the architecture required to form the other junctional complexes^4^. Additionally, E-cadherin has been shown to suppress intracellular signaling pathways, regulating epithelial activation, proliferation and differentiation^3^. We have previously shown that siRNA down-regulation of E-cadherin increases epidermal growth factor receptor (EGFR) activation, inducing expression of the pro-allergic C-C motif ligand 17 (CCL17) and thymic stromal lymphopoietin (TSLP) in human bronchial epithelial cells^5^.

In asthma, the airway epithelial barrier is often compromised, with epithelial denudation, goblet cell metaplasia, ciliary dysfunction and reduced expression of E-cadherin and ZO-1^1,6–10^. This compromised airway epithelial barrier function has already been observed in children with asthma^11^. Asthma is a chronic inflammatory disease of the airways, characterized by eosinophilia, goblet cell metaplasia, airway hyperreactivity and airway remodeling including damage of the airway epithelium. Asthma susceptibility has a genetic component, and the disease is triggered by a hypersensitivity reaction following the interaction of genetic and specific environmental factors, such as aeroallergens, leading to a type-2 immune response.

Aeroallergens are known to directly and indirectly cause disruption of E-cadherin-mediated epithelial junctions^3^. We previously reported that asthma-derived as well as transforming growth factor-beta (TGF)-β-treated airway epithelial cells^12,13^ are more prone to house dust mite (HDM)-induced barrier dysfunction. We further observed that the ability of HDM to induce barrier dysfunction is associated with its ability to induce allergic sensitization and manifestations of asthma *in vivo*^14^. Intranasal HDM exposure has been reported to induce E-cadherin loss *in vivo* and lead to epithelial-to-mesenchymal transition (EMT), a process involved in tissue repair that has been implicated in airway remodeling in asthma^15,16^. It is currently unknown whether the loss of epithelial barrier function in asthma patients is a consequence or cause of the disease.

Since E-cadherin regulates airway epithelial structure, barrier function and innate immune responses^3,5,12,15,16^, we hypothesized that loss of airway epithelial loss of E-cadherin by itself is a critical step leading to the development of asthma manifestations. To test our hypothesis, we generated lung epithelial specific, conditional E-cadherin deficient mice, as germ-line E-cadherin loss has previously been shown to be lethal^17^. We used *sftpc-rtTA/*(tetO)_7_-Cre mice, where pregnant dams were maintained on doxycycline to allow recombination of the conditional allele throughout the conducting airways and parenchyma, as all epithelial cells express surfactant protein C (SFTPC) during early development. Within the airways, SFTPC positive non-ciliated secretory cells called Club cells act as progenitor cells for the goblet and ciliated cells that are responsible for mucociliary clearance^18–20^. Within the alveolar structures, SFTPC positive alveolar type II (ATII) cells serve as progenitors for the alveolar type I (ATI) cells that are responsible for gas exchange^21^. We investigated whether loss of E-cadherin in SFTPC expressing cells during early stages of life is accompanied by an altered airway epithelial phenotype and increased airway inflammation and remodeling later on in life.

## Methods

### Generation of E-cadherin knockout (Cdh1−/−) mice

Conditional E-cadherin knock-out mice (*Cdh1*^fl/fl^, B6.129-Cdh1tm2Kem/J), backcrossed onto the C57Bl/6 J background were purchased from Jackson Laboratory (Bar Harbor, ME). The compound transgenic *sftpc-rtTA/*(tetO)_7_-Cre mice that express the reverse tetracycline trans-activator (rtTA) under control of the rat SFTPC promoter were kindly provided by Prof. Geoffrey Whittset. The SFTPC promoter is expressed as early as day 10 of gestation in epithelial cells of the primordial lung buds^22,23^, causing all lung epithelial cells to express Cre recombinase under control of the tet operator (tetO)^23^. *Cdh1*^fl/fl^ were crossed for two generations with *sftpc-rtTA/*(tetO)_7_-Cre mice to obtain both homozygous *Cdh1*^fl/fl^ Cre^+^ mice (*sftpc-rtTA/(tetO-)*_7_*Cre*^+^*/Cdh1*^*fl/fl*^) and *Cdh1*^fl/fl^ Cre^−^ (*sftpc-rtTA/(tetO)*_7_*-Cre*^−^*/Cdh1*^*fl/fl*^) mice as littermate controls. Pregnant dams were maintained on doxycycline^24^, to allow recombination of the conditional allele in all lung epithelial cells from the earliest developmental stages onward in Cre^+^ progeny. Genotyping was performed as described in the online supplementary information.

Mice were kept under specific pathogen-free conditions and maintained on a 12-hour light-dark cycle, with food and water *ad libitum*. *Cdh1*^fl/fl^ Cre^+^ and *Cdh1*^fl/fl^ Cre^−^ animals were mated and pregnant dams were fed doxycycline-containing chow (200 mg/kg; Bio-Serv, Frenchtown, NJ) until the end of the experiment. Mice were killed by anesthetizing the animals with isoflurane/oxygen (Nicholas Piramal India Ltd., London, UK) at the indicated time points (n = 5–7 mice per group), and bleeding them before removing the lungs. All animal experiments were reviewed and approved by The Institutional Animal Care and Use Committee of the University of Groningen (The Netherlands) and Ghent (Belgium). All experiments were performed in accordance with relevant guidelines and regulations.

### Measurement of airspace enlargement

The mean linear intercept (Lm) was used as morphometric parameter for quantifying airspace size. Histological lung sections, stained with hematoxylin, were imaged using Aperio Scanscope XT and representative samples (three per histological section) were obtained using the non-biased, Systematic Uniform Random Sampling (SURS) method as further detailed in the online supplementary information.

### Immunochemistry, scanning electron microscopy (SEM), flow cytometry and cytokine assays in mouse lung tissue

Lungs were collected for morphometry analysis at day (D)0, week (W)2, W4 and W10 and processed for immunohistochemistry, flow cytometry (only for W2 and W4), see figure S1 in the online data supplement for the gating strategy), SEM, PCR or ELISA (only for W4) as described in the online supplementary information. For the immunohistochemical analysis, staining and number of total airway epithelial cells per length of basement membrane was quantified using Image-Pro Plus. Cells per 500 µm basement membrane (5–25 counts per lung fragment) were counted. Mucus production was assessed by alcian blue staining and goblet cells were stained by Periodic-acid Schiff (PAS).

### Statistics

The non-parametric Mann Whitney U test was performed to assess for significant differences between the Cdh1^fl/fl^ Cre^+^ and Cdh1^fl/fl^ Cre^−^ mice for all analyses. P < 0.05 was considered statistically significant.

## Results

### E-cadherin loss in the airway epithelium causes reduced airway epithelial cell numbers and epithelial denudation

**Figure 1:**
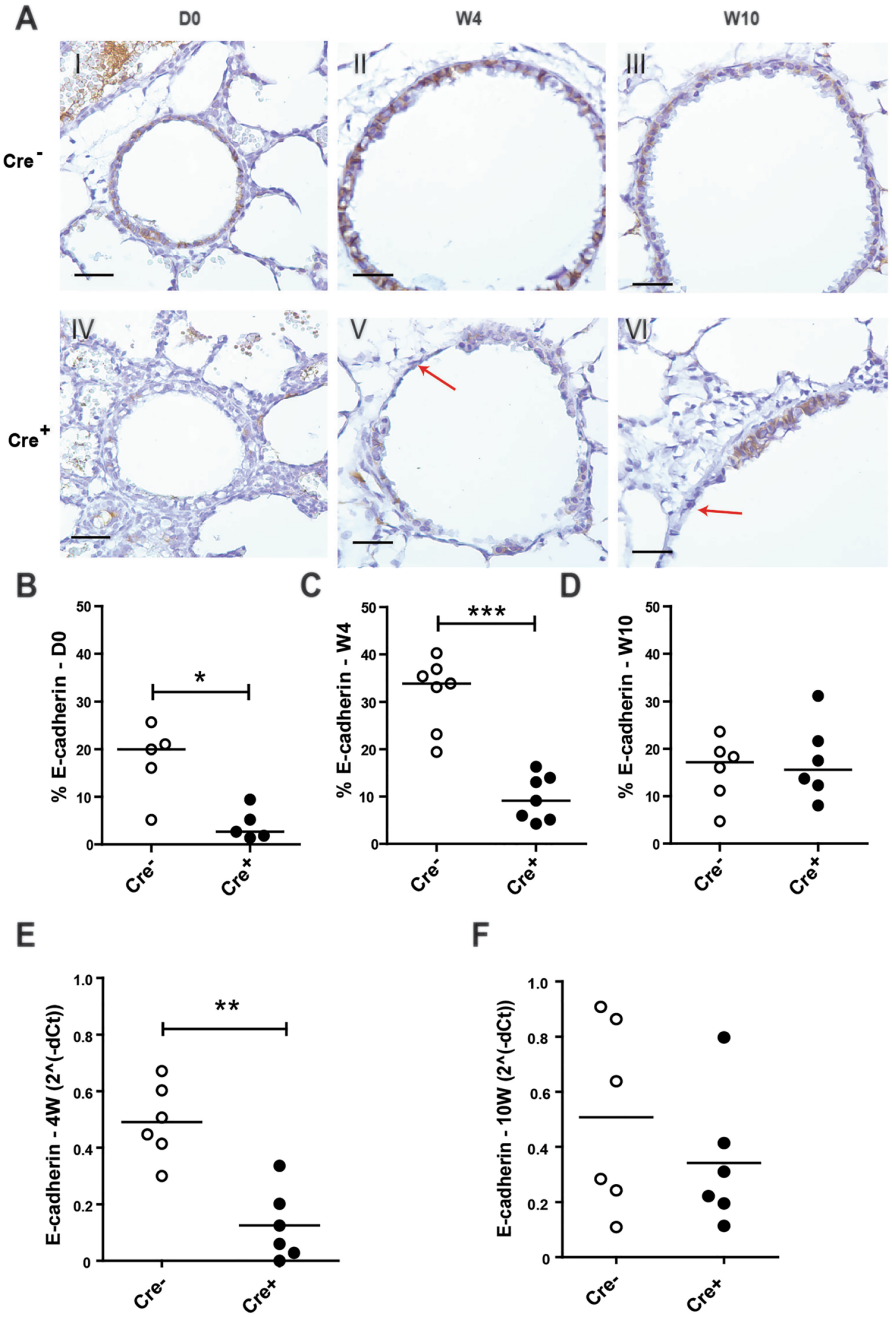
E-cadherin expression in the lungs of E-cadherin knockout (Cdh1^fl/fl^ Cre^+^) and wild type (Cdh1^fl/fl^ Cre^−^) mice (n = 5–7 per group). (**A**) E-cadherin staining of airway epithelium at day (D)0 (I,IV), week (W) 4 (II,V) and W10 (III,VI) of Cdh1^fl/fl^ Cre^−^(I-III)/Cre^+^ (IV-VI) mice. Red arrows indicate epithelial denudation areas. Scale bars: 10 μm. Percentage of E-cadherin positive cell numbers as analyzed by Image-Pro Plus at (**B**) D0, (**C**) W4 and (**D**) W10. mRNA expression of E-cadherin (*cdh1*) in lung homogenates at (**E**) W4 and (**F**) W10. E-cadherin levels were related to the housekeeping genes *hprt1* and *pgk1* and expressed as 2^−ΔCt^. Medians are indicated. *p < 0.05, **p < 0.01 and ***p < 0.001 as assessed by the Mann Whitney U test.

To assess how the loss of E-cadherin within the lung epithelium affects lung development, mice were euthanized on the day of birth (Day (D)0) and at week 4 and 10 of age (W4;W10). At birth, histologically the Cdh1^fl/fl^ Cre^+^ mice displayed normal lung development and the loss of E-cadherin expression in the airway epithelium had no major effect on the development of the bronchi and bronchioles of the lung at this point. Staining for E-cadherin showed presence of E-cadherin in the airway epithelium of Cdh1^fl/fl^ Cre^−^ control littermates at birth, while E-cadherin was significantly reduced and only present in a few scattered airway epithelial cells of the Cdh1^fl/fl^ Cre^+^ mice (Fig. 1A,B). At 4 W, airway epithelial E-cadherin expression remained significantly reduced (Fig. 1A,C), while E-cadherin expression was restored at W10 in the few remaining cells lining the airways (Fig. 1A,D). Accordingly, we observed that E-cadherin mRNA expression was reduced in lung homogenates of Cdh1^fl/fl^ Cre^+^ mice at W4 (Fig. 1E), but not at W10 (Fig. 1F), while we were unable to collect lung tissue for RNA isolation at D0 because of the small size of the lungs.

Interestingly, from the age of W4 onward we observed a damaged airway epithelial layer throughout the lung of the Cdh1^fl/fl^ Cre^+^ mice, with areas showing complete epithelial denudation (Fig. 1A).

We also assessed the effect of E-cadherin loss on tight junction formation by the expression of ZO-1 within the airway epithelium. While there was widespread expression of ZO-1 at D0, by W4 the percentage of positive staining for ZO-1 in *Cdh1*^fl/fl^ Cre^+^ mice was significantly lower compared to control littermates. At W10, ZO-1 expression was no longer significantly decreased in *Cdh1*^fl/fl^ Cre^+^ mice (Fig S1 in the online data supplement).

**Figure 2:**
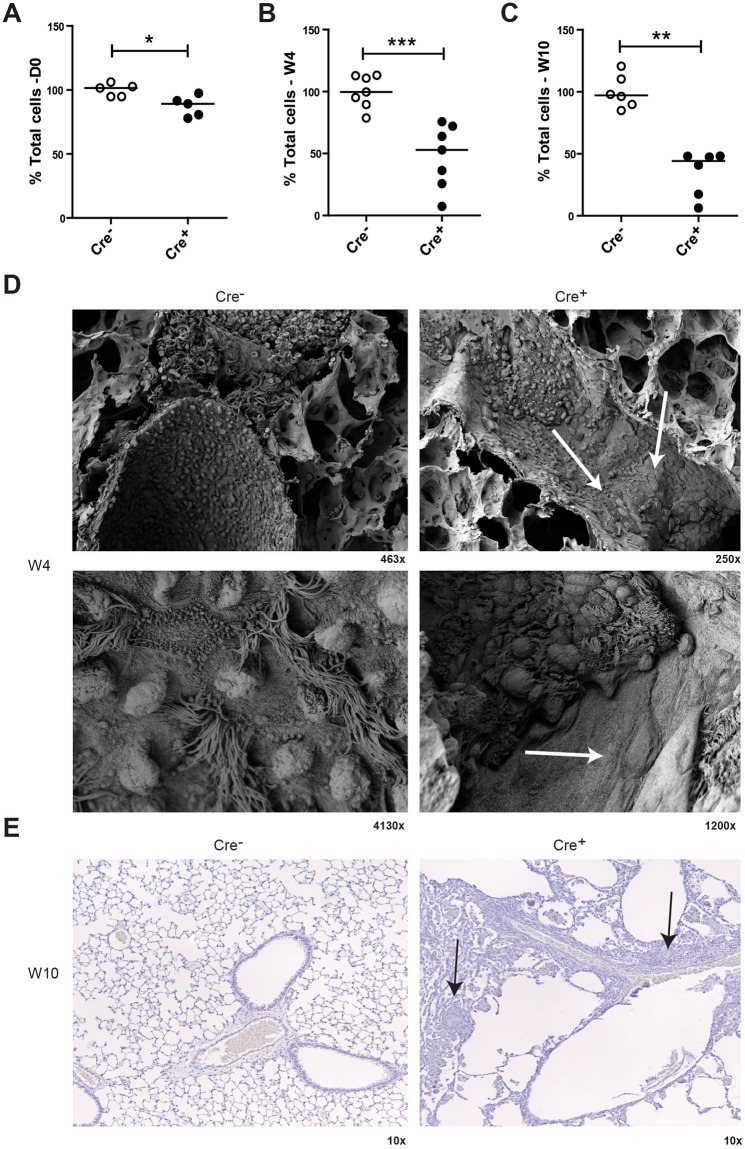
Characterization of airway epithelium in E-cadherin knockout (Cdh1^fl/fl^ Cre^+^) and wild type (Cdh1^fl/fl^ Cre^−^) mice. Analysis of percent total cells in the epithelium of Cdh1^fl/fl^ Cre^−^/Cre^+^ mice at (**A**) day (D)0, (**B**) W4 and (**C**) W10, where the total cell count per 500 µm basement membrane (5–25 counts per lung fragment) per mouse is presented as percentage of the group average. Medians are indicated. (**D**) Electron microscopy images at W4, magnifications are as indicated. White arrows indicate epithelial denudation areas. (**E**) Hematoxylin staining in lung tissue of Cdh1^fl/fl^ Cre^−^/Cre^+^ mice at W8, magnifications are as indicated. *p < 0.05, **p < 0.01 and ***p < 0.001 between the Cdh1^fl/fl^ Cre^+^ and Cdh1^fl/fl^ Cre^−^ mice (n = 5–7 per group) as assessed by the Mann Whitney U test.

Quantification of the number of total airway epithelial cells per length of basement membrane revealed that *Cdh1*^fl/fl^ Cre^+^ mice were in fact born (D0) with significantly reduced airway epithelial cell numbers compared to their control littermates (Fig. 2A), and that the airways of these mice became more denuded at W4 and W10 (Fig. 2B-C). The severity of the epithelial denudation was illustrated by SEM analysis, which revealed almost complete loss of epithelial lining in large regions of the airway lumen in the *Cdh1*^fl/fl^ Cre^+^ mice compared to their control littermates already at the age of W4 (Fig. 2D).

Furthermore, when investigating lung morphology by hematoxylin staining, we observed striking infiltration of inflammatory cells (Fig. 2E).

### E-cadherin deficiency in alveolar type II epithelium leads to increased mean airspace size

**Figure 3:**
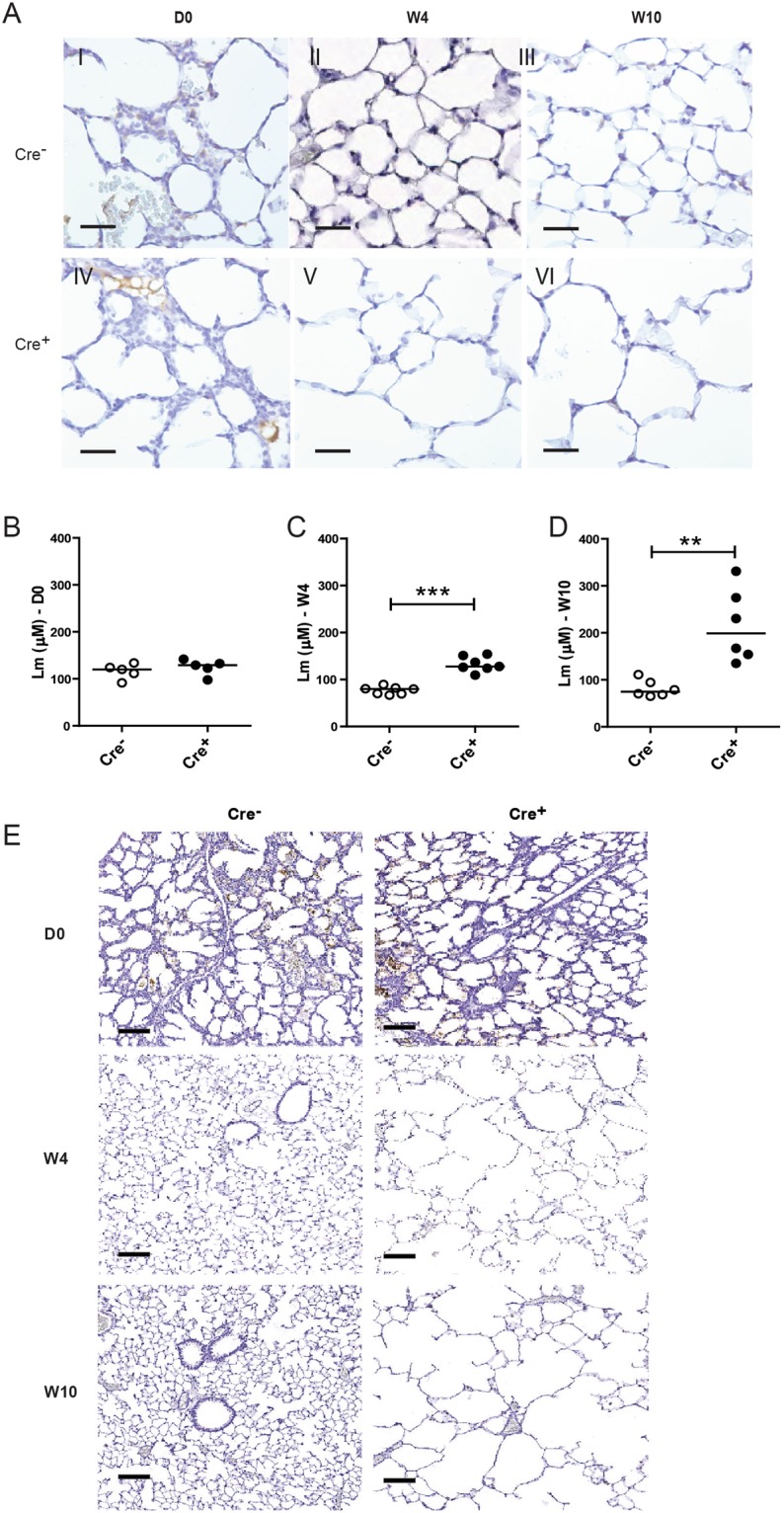
Characterization of alveoli in E-cadherin knockout (Cdh1^fl/fl^ Cre^+^) and wild type (Cdh1^fl/fl^ Cre^−^) mice. (**A**) E-cadherin staining of alveoli at day (D)0 (I,IV), week (W)4 (II,V) and W10 (III,VI) of Cdh1^fl/fl^ Cre^−^ (I-III)/Cre^+^ (IV-VI) mice. Scale bars: 10 μm. Measurement of mean linear intercept (Lm) in the lung tissue at (**B**) D0, (**C**) W4 and (**D**) W10. (**E**) Hematoxylin staining in lung tissue of Cdh1^fl/fl^ Cre^−^/Cre^+^ mice at D0, W4 and W10. Scale bars, 100 μm. An average Lm was calculated from 3 randomly selected regions within each lung. Lm = Number of lines × Length of test line/Number of intersections. A greater Lm value therefore indicates increased air-space size.Medians are indicated. *p < 0.05, **p < 0.001 and ***p < 0.0001 between the Cdh1^fl/fl^ Cre^+^ and Cdh1^fl/fl^ Cre^−^ mice (n = 5–7 per group) as assessed by the Mann Whitney U test.

In addition to the loss of E-cadherin in airway epithelium, we observed E-cadherin loss in ATII cells, which normally express E-cadherin at each time point^25^. This demonstrates that loss of E-cadherin was induced throughout the lung epithelium. Microscopic evaluation of the lung parenchyma revealed that *Cdh1*^fl/fl^ Cre^+^ mice displayed increased mean airspace size (Lm) at W4 and W10 compared to their control littermates (Fig. 3A). *Cdh1*^fl/fl^ Cre^+^ mice demonstrated loss of E-cadherin in all intrapulmonary epithelial cells, including ATII cells (Fig. 3A). Histology analysis and measurement of mean airspace size (Lm) showed that, although *Cdh1*^fl/fl^ Cre^+^ mice had a similar Lm to control *Cdh1*^fl/fl^ Cre^−^ littermates at the time of birth (Fig. 3B), Lm significantly increased in the *Cdh1*^fl/fl^ Cre^+^ mice at W4 and W10 compared to control *Cdh1*^fl/fl^ Cre^−^ littermates (Fig. 3C-D) as further illustrated by HE staining (Fig. 3E). These results indicate that the loss of E-cadherin in ATII cells, the only alveolar cell type to express E-cadherin^25^, affects the overall lung structure inducing emphysematous lesions.

### E-cadherin loss leads to spontaneous loss of ciliated cells and mucus hypersecretion indicating development of goblet cell metaplasia

**Figure 4:**
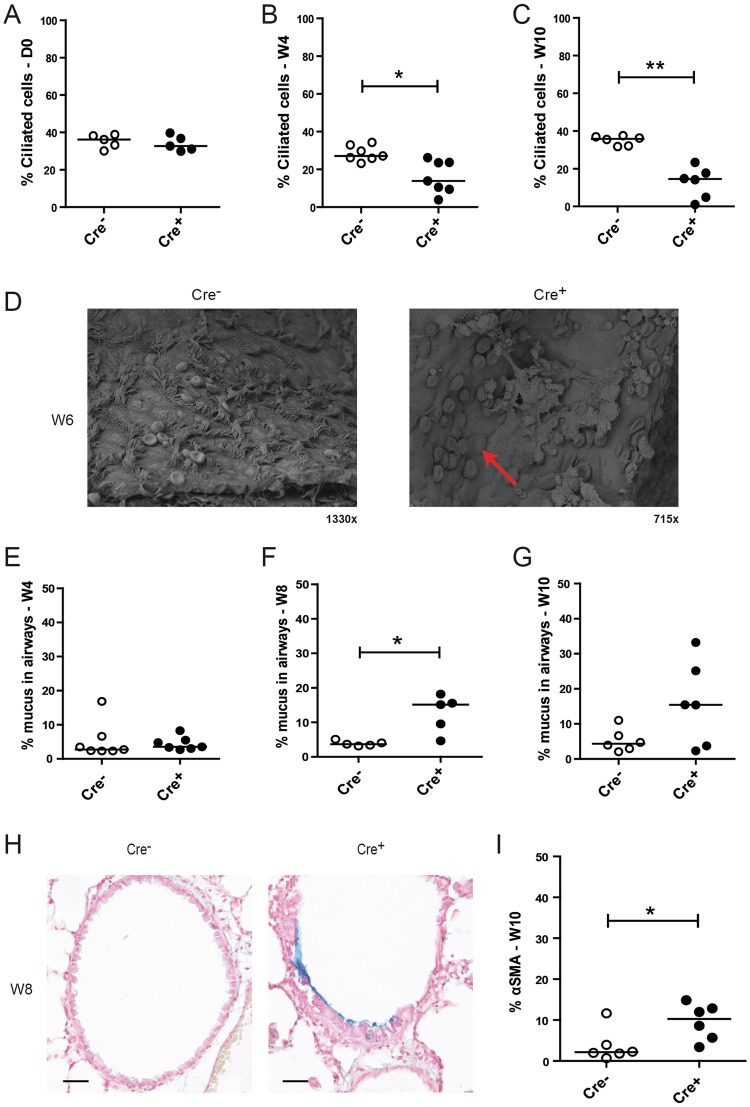
Loss of ciliated cells and increased mucus production in E-cadherin knockout (Cdh1^fl/fl^ Cre^+^) and wild type (Cdh1^fl/fl^ Cre^−^) mice. Analysis of percent ciliated cells at (**A**) day (D)0, (**B**) week (W)4 and (**C**) W10, where the ciliated cell count per mouse was calculated per 500 µm basement membrane (5–25 counts per lung fragment) as percentage of the group average. (**D**) Electron microscopy images at Week W6. Red arrow indicates loss of ciliated cells. Measurement of percentage alcian blue staining in the airways of Cdh1^fl/fl^ Cre^−^/Cre^+^ mice at E) W4, (**F**) W8 and (**G**) W10, (**H**) Representative image of alcian blue staining of Cdh1^fl/fl^ Cre^−^/Cre^+^ mice at W8. Scale bars, 10 μm. (**I**) Percentage of alpha-smooth muscle actin (α-SMA) positive cell numbers as analyzed by Image-Pro Plus in the airways of Cdh1^fl/fl^ Cre^−^/Cre^+^ mice at W10. Medians are indicated. *p < 0.05 and **p < p0.01 between the Cdh1^fl/fl^ Cre^+^ and Cdh1^fl/fl^ Cre^−^ mice (n = 5–7 per group) as assessed by the Mann Whitney U test.

To assess the effect of E-cadherin loss on airway epithelial differentiation, we performed immunohistochemistry staining using the cilia marker acetylated α-tubulin. We observed that acetylated α-tubulin expression was significantly reduced upon E-cadherin loss of in the airways of *Cdh1*^fl/fl^ Cre^+^ mice at W4 and W10 but not at D0, compared to their *Cdh1*^fl/fl^ Cre^−^ control littermates (Fig. 4A–C). This was supported by scanning EM microscopy, revealing that E-cadherin deficiency induced loss of ciliated cell organization within the airways, with remaining ciliated cells scattered in a disorganized fashion along the airways, contrasting the rows of ciliated cells placed at regular intervals along the wild-type control airways (Fig. 4D). In contrast to the observed depletion of ciliated airway epithelial cells, we observed an increase in mucus production, as measured by increased alcian blue staining in *Cdh1*^fl/fl^ Cre^+^ mice at W8 (Fig. 4F–H).

In addition, we assessed the expression of alpha-smooth muscle actin (α-SMA) within the airway epithelium to study if E-cadherin loss induced a more mesenchymal phenotype. We observed an increased expression of α-SMA in E-cadherin deficient airway epithelial cells at age W10, which was not present in the airways of Cre^−^ control littermates (Figure 4JI).

### Loss of E-cadherin results in a pro-inflammatory epithelial phenotype and innate immune activatio

**Figure 5:**
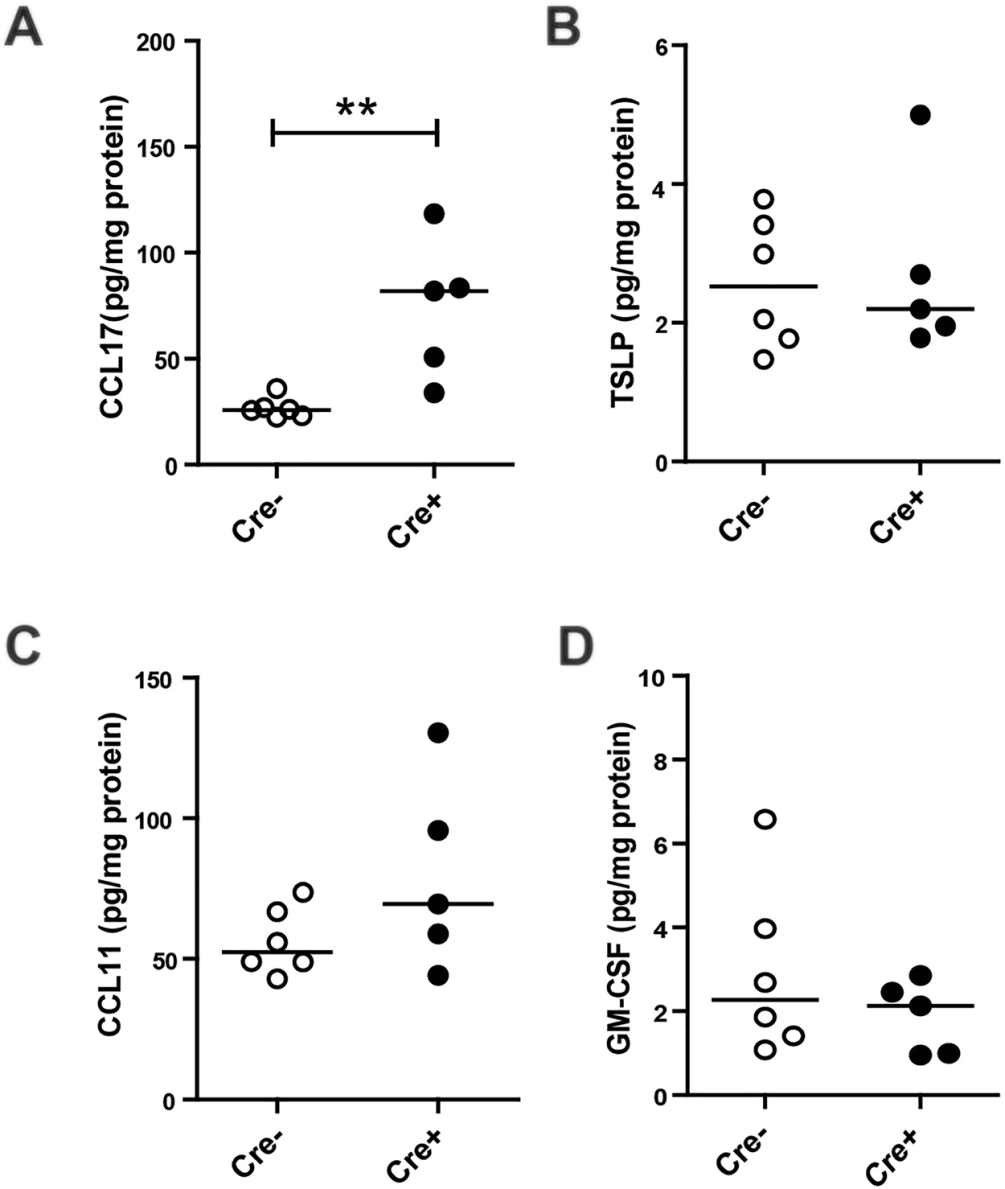
Cytokine responses in E-cadherin knockout (Cdh1^fl/fl^ /Cre^+^) and wild type (Cdh1^fl/fl^ Cre^−^) mice.Enzyme-linked immunosorbent assay (ELISA) analysis for (**A**) Chemokine (C-C motif) ligand 17 (CCL17), (**B**) Thymic stromal lymphopoietin (TSLP), (**C**) C-C motif chemokine 11 (CCL11) and (**D**) Granulocyte-macrophage colony-stimulating factor (GM-CSF) levels in whole lung homogenates at week (W)4. Medians are indicated. **p < 0.01 between the Cdh1^fl/fl^ Cre^+^ and Cdh1^fl/fl^ Cre^−^ mice (n = 5–7 per group) as assessed by the Mann Whitney U test.

As we hypothesized that loss of E-cadherin expression leads to increased production of pro-inflammatory cytokines and activation of an innate immune response, we next investigated the levels of epithelial derived chemokines and recruitment of inflammatory cells into the lung. We observed a significant increase in type-2 T cell attractant CCL17 in *Cdh1*^fl/fl^ Cre^+^ mice compared to *Cdh1*^fl/fl^ Cre^−^ mice when mice reached the age of W4 (Fig. 5A), while levels of TSLP, C-C motif chemokine 11 (CCL11) and granulocyte-macrophage colony-stimulating factor (GM-CSF) were not different between the groups (Fig. 5B–D).

**Figure 6:**
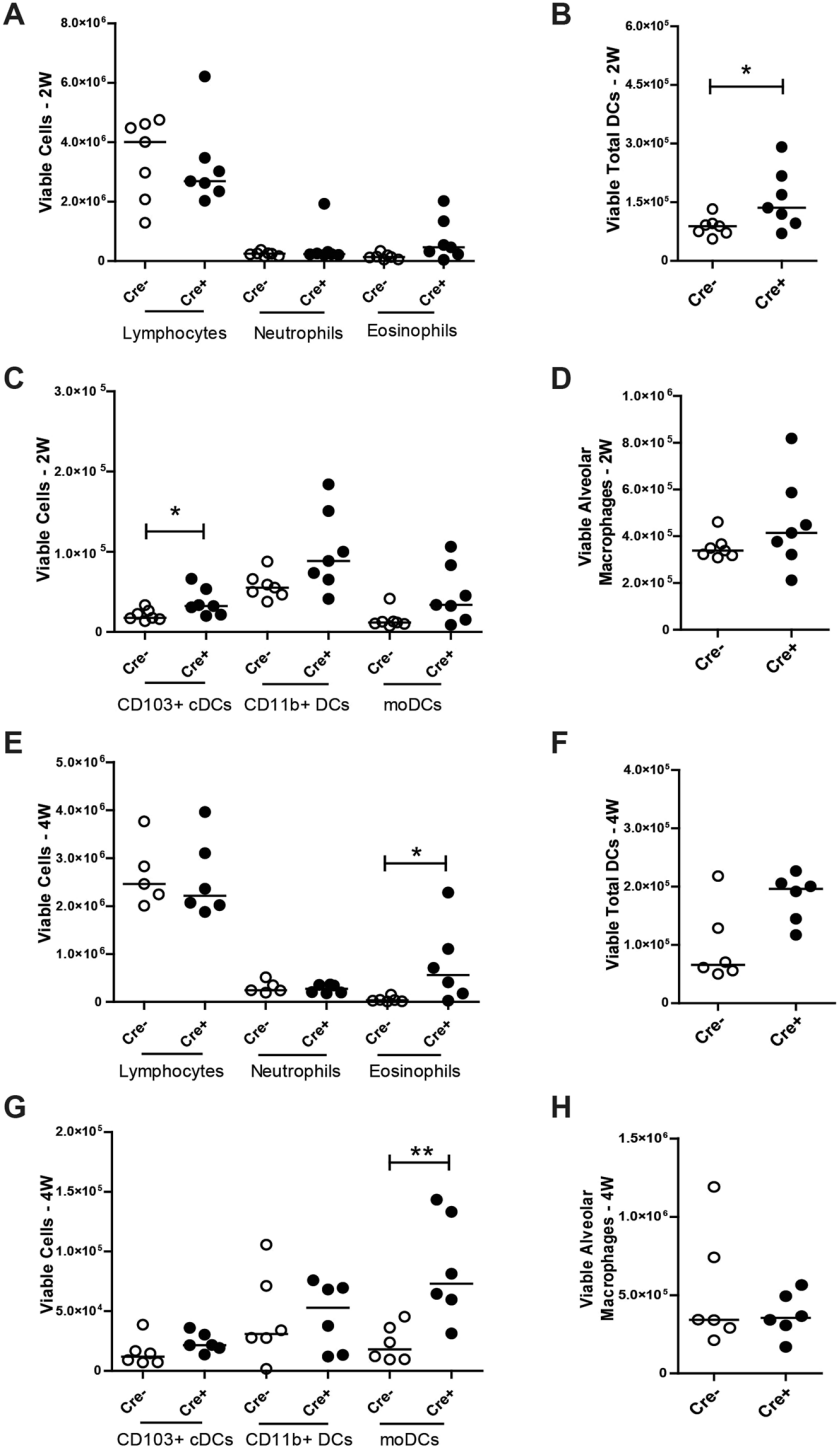
Inflammatory responses in E-cadherin knockout (Cdh1^fl/fl^ Cre^+^) mice) and wild type (Cdh1^fl/fl^ Cre^−^) mice. Flow cytometry analysis for inflammatory cells in whole lungs of Cdh1^fl/fl^ Cre^−^/Cre^+^ mice at (**A**) week (W)2 and (**E**) W4. Dendritic cell (DC) populations at (**B**) W2 and **F**) W4 and DC sub-populations (CD103^+^ conventional (c**)**DCs, CD11b + DCs and monocyte-derived (mo)DCs) at (**C**) W2 and (**G**) W4. Alveolar macrophages at (**D**) 2 W and (**H**) 4 W. Medians are indicated. *p < 0.05, **p < 0.01 or p value is as indicated between the Cdh1^fl/fl^ Cre^+^ and Cdh1^fl/fl^ Cre^−^ mice (n = 5–7 per group) as assessed by the Mann Whitney U test.

Next, we performed a detailed flow cytometric characterization of the infiltrated inflammatory cells observed by hematoxylin staining (Fig. 2E) in the lung tissue at W2 and W4 (Fig. 6A–H). We found that eosinophil numbers increase as early as at W2 in the lungs of *Cdh1*^fl/fl^ Cre^+^ mice (Fig. 6A) compared to *Cdh1*^fl/fl^ Cre^−^ mice. This increase reached statistical significance at W4 (Fig. 6E). Additionally, we observed that the total dendritic cell (DC) population was significantly increased in the lungs of the *Cdh1*^fl/fl^ Cre^+^ mice compared to their control littermates at W2 (Fig. 6B), but no longer at W4 (Fig. 6F). Interestingly, phenotyping the DC subpopulations at W2 showed that the CD103^+^ conventional (c)DC population was significantly elevated in *Cdh1*^fl/fl^ Cre^+^ lungs compared to *Cdh1*^fl/fl^ Cre^−^ lungs (Fig. 6C), while the inflammatory monocyte-derived DCs (moDCs) were significantly elevated in the *Cdh1*^fl/fl^ Cre^+^ lungs at W4 (Fig. 6G). On the other hand, no effect was observed for CD11b^+^ cDCs at either 2 W or 4 W (Fig. 6C,G). Furthermore, total lymphocyte, neutrophil and macrophage numbers in the lungs were not affected by E-cadherin deficiency at any age (Fig. 6A,D,E,H). Together, these data show that E-cadherin deficiency leads to pro-inflammatory activation of the lung epithelium that specifically attracts eosinophilic granulocytes and inflammatory DCs into the airways.

## Discussion

We show for the first time that conditional loss of E-cadherin results in airway epithelial denudation, loss of ciliated cells, spontaneous induction of mucus hypersecretion indicating goblet cell metaplasia and eosinophilic inflammation, all characteristics of asthma. Conditional loss of E-cadherin in the lung epithelium did not affect lung development up to birth. However, as the Cre^+^ mice aged, they developed airspace enlargement characteristic of emphysematous lesions associated with loss of E-cadherin in ATII cells.

Our current study supports the hypothesis that loss of epithelial E-cadherin as observed in asthma has important consequences, contributing to the pathogenesis of asthma, integrating structural and immunological regulatory functions within the airway epithelium^3^. Although E-cadherin is known to be critical for organogenesis of several epithelial tissues^17,26^, we observed a roughly normal lung anatomy at the time of birth in the *Cdh1*^fl/fl^ Cre^+^ mice, with only very few epithelial cells expressing E-cadherin. This might be explained by the timing of E-cadherin loss in our model. A previous study showed that SFTPC-CAT expression at high levels was first detected as early as day 10 of gestation (E10) in epithelial cells (both airway epithelial cells and ATII cells) of the primordial lung buds^23^. Therefore, we anticipate that E-cadherin deficiency would not be introduced until the second week of gestation (~E10), when the primary lung epithelium has already formed, allowing normal development of the lung. Nonetheless, SFTPC-driven Cre expression during embryonic development has previously been shown to induce recombination in all epithelial cells contributing to lung development, and was passed on to all lung epithelial cell into adulthood^23^. These data may explain our observation of normal lung development at birth, even with almost complete E-cadherin loss in the lung epithelium.

The lung morphology markedly changed when *Cdh1*^fl/fl^ Cre^+^ mice reached an adult age, confirming that E-cadherin is a central regulator of lung structure and inflammation. Its loss resulted in epithelial denudation, decreased ZO-1 expression, loss of ciliated cell numbers and organization, goblet cell metaplasia, and inflammatory cell infiltration in the conducting airways as well as enlarged airspace size. These data are in line with a recent study demonstrating that E-cadherin is necessary for the differentiation of Club cells^27^, acting as progenitor cells for ciliated cells^28^. Postnatal inactivation of E-cadherin in mice impaired the repair of the conducting airway epithelium after site-specific Club cell injury^27^. We anticipate that a repair response is provoked in Cre^+^ mice upon the loss of E-cadherin, requiring proliferation and re-differentiation of Club cells progenitors to reconstruct a polarized, functionally intact ciliated epithelial layer. Lack of this process in *Cdh1*^fl/fl^ Cre^+^ mice could be a consequence of E-cadherin down-regulation in Club cells. Of note, basal side population cells that express breast cancer resistance protein (BCRP1), cytokeratin (CK)5 and p63 have been proposed as a major airway epithelial stem cell involved in repair of the conducting airways^8^. These BCRP1^+^CK5^+^p63^+^ cells are also capable of generating Club cell progenitors, but do not express transcription factors such as SFPTC, which are turned on later during development or regeneration. Therefore, these side population cells may be responsible for the recurrence of E-cadherin expressing cells in the bronchial epithelial lining of *Cdh1*^fl/fl^ Cre^+^ mice at adult age, since these and their daughter cells are not affected by doxycycline-induced E-cadherin deficiency. Additionally, epithelial injury has been shown to induce Club cell metaplasia into mucus-producing cells in order to restore the damage^20^. Our data suggest that E-cadherin deficiency results in spontaneous goblet cell metaplasia, indicative of mucus hypersecretion by Club cells. In addition, we observed α-SMA expression in the epithelial layer, which could reflect a repair mechanism involving the transition to a more mesenchymal phenotype (e.g. EMT). In line, postnatal inactivation of E-cadherin was previously shown to induce WNT/β-catenin signaling, an important component of EMT, and bronchiolar metaplasia^27^. Expression of activated β-catenin in airway epithelium may not only result in EMT-like features, but has also been shown to result in goblet cell metaplasia through a mechanism involving down-regulation of mucus repressor Foxa2^29^. Thus, the spontaneous goblet cell metaplasia in the *Cdh1*^fl/fl^ Cre^+^ mice could be explained by dysregulated β-catenin signalling and abnormal repair responses. Another mechanism that could be involved in the observed mucus hypersecretion upon E-cadherin deficiency may be an increase in EGFR signalling, as we previously observed that loss of E-cadherin expression by airway epithelium *in vitro* leads to increased EGFR signalling^30^, which has been implicated in mucus production^31^. E-cadherin deficiency also resulted in decreased tight junctional protein ZO-1, which is in line with previous studies that have shown that loss of E-cadherin impairs formation of tight junctions, leading to barrier dysfunction and reduced expression of ZO-1^3^. These data along with studies in asthmatic airway epithelial biopsies that have previously shown decreased E-cadherin and ZO-1 expression^6^ further support the role of E-cadherin loss in compromised airway epithelial barrier function in asthma.

Alongside the airway epithelial changes, lungs of *Cdh1*^fl/fl^ Cre^+^ mice showed enlarged airspaces with thinned and damaged septa, which became more pronounced with age. While asthma is considered mainly as an airway obstructive lung disease, a limited number of studies demonstrated parenchymal abnormalities in asthma patients, including centrilobular micronodules, mosaic perfusion and increased percent lower attenuation areas related to emphysematous changes^32,33^. In the parenchyma, specifically ATII cells express SFTPC, thus the SFTPC promotor ensured E-cadherin deficiency in ATII cells in our model. ATII cells function as a progenitor for alveolar type I epithelium in rodents and humans^34,35^. In addition, ATII cells are responsible for the production of pulmonary surfactant, which is required for epithelial integrity, adapting to breathing after birth by reducing surface tension in the alveolus^36^. Disruption of E-cadherin in mature ATII cells was previously shown to lead to diffuse hyperplasia and airspace enlargement^37^. Similarly, expression of activated β-catenin and of Foxa2 expression in mouse lungs resulted in aberrant ATII differentiation and airspace enlargement^29,38^. No formal study has investigated whether the mechanism underlying the emphysematous changes in E-cadherin deficient mice involves loss of SFTPC^+^ATII cells or deregulated β-catenin signaling, needing further investigation.

In addition to direct effects on airway epithelium, we observed a striking pro-inflammatory response of the airway epithelium, as evidenced by the production of CCL17, finally resulting in airway inflammation characterized by eosinophils and inflammatory dendritic cells. We have previously shown that down-regulation of E-cadherin expression in bronchial epithelial cells results in increased EGFR-induced expression of CCL17 and TSLP^39^. Additionally, E-cadherin loss may lead to activation of the nuclear factor-kappa B (NF-κB) and MAPK signaling pathways^40^. Both EGFR and NF-κB-mediated signaling can induce secretion of pro-inflammatory cytokines and chemokines, including CCL17, suggesting that the induction of E-cadherin loss by itself is sufficient to induce pro-inflammatory activity of the airway epithelium^12^. In this way, loss of E-cadherin could lead to type-2 T cell-mediated eosinophilia. Alternatively, type-2 innate lymphocyte cells could be responsible for the observed eosinophils^41^, but this requires further investigation and will be subject of future studies. Of interest, loss of Foxa2 expression in lung epithelium in a mouse model not only induced goblet cell metaplasia, but also spontaneous pulmonary eosinophilic inflammation, recruitment of mDCs and type-2 T cells and increased levels of various chemokines, including CCL17^42^. As Foxa2 is known to activate the E-cadherin promoter^43^, loss of E-cadherin might play a central role in this phenotype given the striking similarities with our model. In addition, to the lung epithelium, CCL17 can be produced by CD103^+^ cDC and moDCs populations, which play a predominant pro-inflammatory role in the development of asthma^44^. We observed significantly elevated levels of both CD103 (α_E_ß_7_ integrin)^+^ cDC and moDCs in the lungs of the young adult *Cdh1*^fl/fl^ Cre^+^ mice compared to their littermate controls. This may contribute to innate immune activation in the E-cadherin-deficient mice.

Our results in this novel mouse model of engineered loss of epithelial integrity strongly suggest that E-cadherin delocalization by itself may be sufficient for the development of airway inflammation. In line, structural changes in the airway epithelium as well as airway eosinophilic inflammation have been observed in the lungs of children before allergic asthma was even diagnosed^45^. In addition, a study of Ierodiakonou *et al*. showed that *CDH1* gene polymorphisms are associated with airway remodeling and inflammation as well as lung functions in asthmatic patients^46^, but only in patients using inhaled corticosteroids. Collectively, these studies and our data suggest that disruption of epithelial cell-cell contacts with junctional loss of E-cadherin are a crucial event in the development of asthma. In future studies, it will be of interest to assess whether the loss of E-cadherin also increases allergic sensitization.

Taken together, our data show that loss of E-cadherin in lung epithelia induces spontaneous changes that have remarkable characteristics of an asthmatic phenotype, including progressive loss of airway epithelial cells, spontaneously mucus hypersecretion and eosinophilic airway inflammation. This indicates that loss of E-cadherin in the airway epithelium is not merely a consequence of disease, but actively contributes to the pathogenesis of asthma, identifying E-cadherin as a novel target for future therapeutic strategies.

## Electronic supplementary material

Supplementary Data

## Data Availability

The datasets generated during the current study are available from the corresponding author on reasonable request.
