# Supplementary material for: Characterization of a lung epithelium specific E-cadherin knock-out model: Implications for obstructive lung pathology
Source: Sci Rep. 2018 Sep 5;8:13275. doi: 10.1038/s41598-018-31500-8 (PMC6125431; doi:10.1038/s41598-018-31500-8)
Supplement: Supplementary file 1 — Supplementary Data [file 41598_2018_31500_MOESM1_ESM.docx]

**Online supplementary information**

**Characterization of a lung epithelium specific E-cadherin knock-out model: Implications for obstructive lung pathology**

S. Post^1,2,4^*, I.H. Heijink^1,2,3,^*, L. Hesse^1,2^, H.K. Koo^4^, F. Shaheen^4^, M. Fouadi^4^, V.N.S. Kuchibhotla^1,2^, B.N. Lambrecht^5,6,7^, A.J.M. van Oosterhout^1,2^, T.L. Hackett^4,#^, and M.C. Nawijn^1,2,#^

**Affiliations**

^1^ University of Groningen, University Medical Center Groningen, Department of Pathology & Medical Biology, laboratory of Experimental Pulmonology and Inflammation Research (EXPIRE), Groningen, The Netherlands.

^2^ University of Groningen, University Medical Center Groningen, GRIAC Research Institute, Groningen, the Netherlands.

^3^ University of Groningen, University Medical Center Groningen, Department of Pulmonology, Groningen, the Netherlands

^4^ University of British Columbia, Centre for Heart and Lung Innovation, Department of Anesthesiology, Pharmacology and Therapeutics, St. Paul’s Hospital, Vancouver, British Columbia, Canada

^5^ Laboratory of Immunoregulation and Mucosal Immunology, Department for Molecular Biomedical Research, Inflammation Research Centre (IRC), Ghent, Belgium

^6^ Department of Pulmonary Medicine, Ghent University, Ghent, Belgium

^7^ Department of Pulmonary Medicine, Erasmus University Medical Center Rotterdam, Rotterdam, the Netherlands

* These authors contributed equally to the manuscript.

^#^ These authors share senior-authorship.

**Methods**

***Genotyping***

Mice were identified using PCR primers specific for each promoter (E-cadherin; fwd: GGG TCT CAC CGT AGT CCT CA, E-cadherin rvs: GAT CTT TGG GAG AGC AGT CG; SP-C-rtTA fwd: AAA AT CT TG CCA GC TT TC CCC, SP-C-rtTA rvs: ACT GC CC AT TGC CC AA AC AC; Cre fwd GC CA CG AC CAA GT GA CA GCA AT G, Crw rvs: AGA GA CG GA AAT CC AT CG CTC G). PCR analysis was performed on tissue lysates of the mouse tails. Amplification of PCR products for E-cadherin, *sftpc*-rtTA and Cre were performed as following: Denaturation at 95°C for 5 min, 35 cycles denaturation at 95°C for 30 s, annealing at 62°C for 1 min, and extension at 72°C for 1 min, followed by 10 extensions at 72°C.

***Isolation of lung cells and flow cytometry analysis***

Mice were sacrificed at W2 and W4 and part of the lung was collected and digested using Roswell Park Memorial Institute (RPMI, Sigma) medium containing Liberase TM (Roche, Basel, Switserland) and 10U DNAse (Roche). The cell suspension was lysed for 4 minutes in 1 ml osmotic lysis buffer at room temperature and the antibody staining reactions were performed at 4°C for 30 to 45 min. Lung cells were incubated with 2.4G2 Fc receptor Ab to reduce non-specific binding, and then stained with monoclonal antibodies directed against lymphocyte antigen 6 complex (Ly6C; AL21), Siglec F (E50-2440), CD64 (X54-5/7.1) and CD11b (M1/70) (BD Bioscience), CD3 (145-2C11), CD19 (1D3), CD11c (N418), Ly6G (1A8), CD103 (2E7) and SAV (eBioscience), major histo-compatibility complex class 2 (MHCII; M5/114; Biolegend), and a fixable live/dead marker Aqua (Invitrogen). Acquisition of the 11-color samples was done on a LSR Fortessa cell analyzer cytometer. Final analysis and graphical output were performed by using FlowJo software (Treestar, Costa Mesa, CA.).

***Scanning Electron Microscopy (SEM)***

### **Lungs used for the electron microscopy analyses were cut in 5 mm thin slices. Briefly, the lung samples were fixated in** 2.5% glutaraldehyde in 0.1M cacodylate buffer, pH 7.4 at 4° C overnight, followed by washes in 0.1 M cacodylate buffer (pH 7.4) for 5 minutes for each wash. Lung samples were then immersed in 1% osmium tetroxide (pH 7.4) for 1 hour at room temperature followed by 3 washes in 0.1 M cacodylate buffer (pH 7.4) for 5 minutes for each wash. After which the lung samples were dehydrated in various concentrations of ethanol; 25% (10 minutes), 50% (10 minutes), 70% (10 minutes), 85% (10 minutes), 95% (10 minutes), 100% (2x 10 minutes), 100% (10 minutes). After the dehydration the dried lung samples were mounted onto a metal stub with double sided carbon tape and coated with a thin layer of gold and palladium using an automated sputter coater. The mounted lung pieces were than imaged using a Merlin FE-SEM with 3D View2 XP Gatan from Zeiss.

***Histology and immunochemistry***

Lungs were taken for morphometry analysis at W0, W4 and W10 and prepared for histological analysis as previously described^1,2^. Briefly, mice were sacrificed on day (D) 0, W4, W8 and W10, and lungs were inflated with TissueTek O.C.T. Compound (Sakura Finetek Europe B.V, Zouterwoude, The Netherlands), and fixed in 10% Formalin for 24 hours, embedded in paraffin and cut in 5 μm-thick sections. Lung sections were stained with hematoxylin and alcian blue.

For the hematoxylin staining, lung sections were de-paraffinized, rehydrated in ethanol and washed in PBS. The lung sections were then incubated with Harris-Hematoxylin (Sigma, Zwijndrecht, Netherlands) for 1 minute and washed in tap water, followed by dehydration with ethanol and cover slipped using cytoseal^TM^60 (**Thermo Fisher Scientific, New Jersey, USA).**

**For the alcian blue staining,** lung sections were de-paraffinized, rehydrated in ethanol and washed in PBS. After the wash, lung sections were incubated with alcian blue solution pH 2.5 for 30 minutes, followed by washing with running tap water. Lung sections were then counterstained in 0.1% nuclear fast red solution for 5 minutes, followed by another wash with running tap water. At the end, the lung sections were dehydrated with ethanol and coverslipped using cytoseal^TM^60 **(Thermo Fisher Scientific).**

For staining of E-cadherin (BD Biosciences, Erembodegem, Belgium), acetylated alpha tubulin (Abcam, Cambridge, UK) and zonula occludens (ZO)-1 (Abcam, ab216880, Cambridge, UK) lung sections were de-paraffinized in xylene, rehydrated in ethanol and washed in TBS. Antigen retrieval was performed by heating lung sections to boiling point in Target Retrieval Solution Citrate at pH 6.0 (DAKO, Glostrup, Denmark) for 15 minutes for E-cadherin and acetylated alpha tubulin staining. For ZO-1, antigen retrieval was performed with Envision FLEX target antigen retrieval solution (DAKO) in a water bath for 10 min at 90-95 °C. Sections were then washed with TBS and blocked with TBS containing 30% H_2_O_2_ (Thermo Fischer Scientific) for 30 min, followed by a block with horse serum (50%; Vector Laboratories inc., Burlingame, CA) for 1 hour. Lung sections were immunostained with mouse-anti-E-cadherin (1/400), mouse-anti-acetylated alpha tubulin (1/200) or rabbit-anti-ZO-1 (1/250) for 1 hour, followed by incubation with the secondary Ab (1/400), biotinylated-anti-mouse IgG (horse) or biotinylated-anti-rabbit IgG (goat) containing 10% albumin from bovine serum (BSA; Sigma) for 30 minutes. After the second Ab incubation, lung sections were washed with TBS and incubated with Streptavidin/HRP (1/1000; DAKO) for 15 minutes. The immunostains were developed by using 3,3'-diaminobenzidine (DAB; DAKO) substrate and counterstained with Harris-Hematoxylin (Sigma), followed by cover-slipping using cytoseal^TM^60 (**Thermo Fisher Scientific).**

Images were acquired using a Nikon eclipse C600 microscope attached to a Color digital camera (Diagnostic Instrument Inc., Sterling Heights, Michigan) using SPOT software (version 4.6; Diagnostic Instrument Inc.). Staining and number of total airway epithelial cells per length of basement membrane was quantified using Image Pro Plus^1^.

***Measurement of airspace enlargement***

The mean linear intercept (Lm) was used as morphometric parameter for quantifying airspace size. Histological lung sections, stained with hematoxylin, were imaged using Aperio Scanscope XT and representative samples (three per histological section) were obtained using the non-biased, Systematic Uniform Random Sampling (SURS) method as further detailed in the online data supplement.. A standardized 4x4 grid mask with 36 test lines of equal length was applied to each histological sample using ImagePro with a resolution of 1.004 microns/pixel. Lm was measured by counting the number of alveolar septal intersections through each grid line. Any test line with intercepts over an airway, vessel or artifact, were excluded. Lm was calculated using the following formula: *Lm = Number of lines x Length of test line/Number of intersections.* A greater Lm value therefore indicates increased air-space size.

### *Cytokine assay in mouse lung tissue*

### Levels of CCL17, TSLP, eotaxin-1 and GM-CSF were determined in homogenized lung tissue lysates of W4 using Duoset ELISA Development Kit’s (R&D Systems, Minneapolis, MN), according to the manufacturer’s guidelines.

***PCR in mouse lung tissue***

E-cadherin expression was determined in homogenized lung tissue lysates upon RNA isolation using TRIreagent (MRC, Cincinnati, OH). Samples were treated with RNAse Free DNAse and subsequently cleaned with RNeasy Mini Kit (Qiagen). cDNA was synthesized with the iScript cDNA Synthesis Kit (BioRad). Gene expression was analyzed by real-time PCR using the Taqman® according to the manufacturer’s guidelines (Applied Biosystems, Foster City, CA). Validated probes for E-cadherin (*cdh1*) and the housekeeping genes *hprt1* and *pgk1* were purchased from Applied Biosystems. The qPCR Master mix was purchased from Promega (Madison, WI).

**
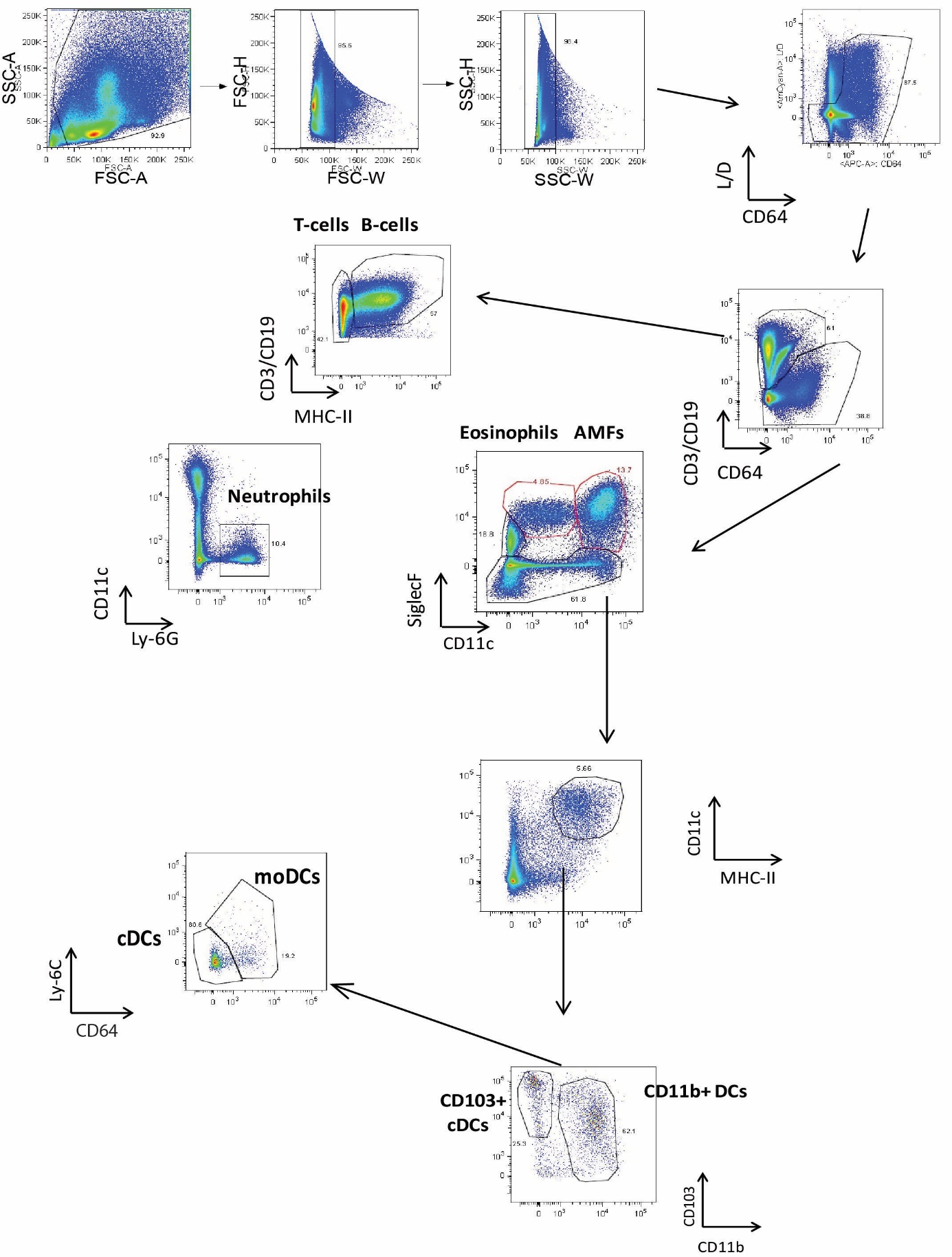
**

**Figure S1. Gating strategy for the flow cytometry on inflammatory cells in lung tissue.** Flow cytometry analysis for macrophages, lymphocytes (T-cell, B-cells), granulocytes (neutrophils, eosinophils) and dendritic cells (conventional (c)DCs, CD11b+ DCs and monocyte-derived (m)DCs).


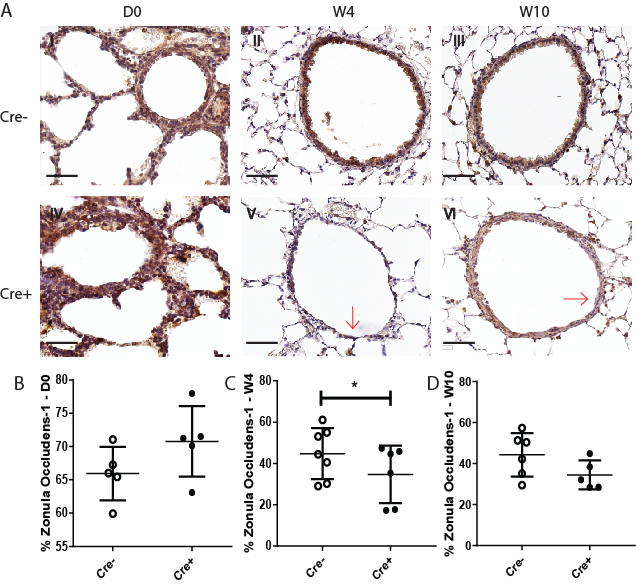


**Figure S2. Zonula occludens-1 expression in the lungs of E-cadherin knockout (Cdh1^fl/fl^ Cre^+^) and wild type (Cdh1^fl/fl^ Cre^-^) mice (n=5-7 per group).**

A) Zonula Occludins-1 staining of airway epithelium at day (D)0 (I,IV), week (W) 4 (II,V) and W10 (III,VI) of Cdh1^fl/fl^ Cre^-^(I-III)/Cre^+^ (IV-VI) mice. Red arrows indicate epithelial denudation areas. Scale bars: 10 μm. Percentage of zonula occludens-1 positive cell numbers as analyzed by Image-Pro Plus at B) D0, C) W4 and D) W10. *=p<0.05 as assessed by the Mann Whitney U test.

**References**

1 Post S, Nawijn MC, Hackett TL, Baranowska M, Gras R, van Oosterhout AJ *et al.* The composition of house dust mite is critical for mucosal barrier dysfunction and allergic sensitisation. *Thorax* 2012; **67**: 488–495.

2 Hacket TL, Ferrante SC, E HC, Engelhardt JF, Ingram JL, Yulong Z *et al.* A heterotopic xenograft model of human airways for investigating fibrosis in asthma. *Am J Respir Cell Mol Biol* 2016. doi:10.1165/rcmb.2016-0065MA.
